# Healthcare access: A sequence-sensitive approach

**DOI:** 10.1016/j.ssmph.2016.11.008

**Published:** 2016-11-30

**Authors:** Marco J. Haenssgen, Proochista Ariana

**Affiliations:** a Centre for Tropical Medicine and Global Health, Nuffield Department of Medicine, University of Oxford, Old Road Campus, Roosevelt Drive, Oxford OX3 7FZ, UK; b CABDyN Complexity Centre, Saïd Business School, University of Oxford, Park End Street, Oxford OX1 1HP, UK; c Technology and Management Centre for Development, Oxford Department of International Development, University of Oxford, 3 Mansfield Road, Oxford OX1 3TB, UK; d Green Templeton College, 43 Woodstock Road, Oxford OX2 6HG, UK; e Economics and Translational Research Group (ETRG), Mahidol Oxford Tropical Medicine Research Unit (MORU), Faculty of Tropical Medicine, Mahidol University, 3/F, 60th Anniversary Chalermprakiat Building, 420/6 Rajvithi Road, Bangkok, Thailand

**Keywords:** India, China, Healthcare-seeking behavior, Sequence analysis, Methodology, Rural survey

## Abstract

It is widely accepted that healthcare-seeking behaviour is neither limited to nor terminated by access to one single healthcare provider. Yet the sequential conceptualisation of healthcare-seeking processes has not diffused into quantitative research, which continues to analyse healthcare access as a “one-off” event. The ensuing lack of understanding healthcare behaviour is problematic in light of the immense burden of premature death especially in low- and middle-income countries. This paper presents an alternative approach. Based on a novel survey instrument, we analyse original survey data from rural India and China that contain 119 unique healthcare pathways among 637 respondents. We offer three applications of how such sequential data can be analysed to enhance our understanding of people's health behaviour. First, descriptive analysis of sequential data enables more a comprehensive representation of people's health behaviours, for example the time spent in various healthcare activities, common healthcare pathways across different groups, or shifts in healthcare provider access during a typical illness. Second, by analysing the effect of mobile technology on healthcare-seeking process characteristics, we demonstrate that conventional, sequence-insensitive indicators are potentially inconsistent and misleading approximations when compared to a more precise, sequence-sensitive measure. Third, we describe how sequential data enable transparent and flexible evaluations of people's healthcare behaviour. The example of a sequence-insensitive evaluation suggests that household wealth has no statistical link to an illustrative “ideal” form of public healthcare utilisation. In contrast, sequence-sensitive evaluations demonstrate that household wealth is associated with an increased likelihood of bypassing referral processes and approaching unregulated and costly informal and private practitioners before accessing a public clinic. Sequential data therefore do not only reveal otherwise neglected locational idiosyncrasies, but they also yield deeper insights into the drivers of people's health behaviours compared to a conventional approach to “access to healthcare.”

## Highlights

• Despite its sequential nature, healthcare seeking is often analysed as single event.
• We demonstrate the value of sequential healthcare data analysis.
• Descriptive analysis exposes otherwise neglected behavioural patterns.
• Sequence-insensitive indicators can be inconsistent and misleading.
• Sequence-sensitive evaluation hints at adverse behaviours of wealthy patients.

## 1 Introduction

People in low- and middle-income countries die on average more than 20 years younger than high-income-country citizens (Institute for Health Metrics and Evaluation, 2012, World Bank, 2015). In the context of the 2030 Sustainable Development Goals, the current Universal Health Coverage agenda calls for widespread and timely access to healthcare to relieve poor households in low- and middle-income countries from this disease burden and from associated catastrophic health expenditures (Summers, 2015). However, access to healthcare is not a straightforward concept because people's health decisions are often subject to constraints like poverty, access to finance, time restrictions, or lack of quality healthcare providers. Healthcare-seeking processes under such constraints can result in various combinations of “no care,” “self-care,” and healthcare from many different practitioners (Kibadi et al., 2009, MacKian et al., 2004; Nyamongo, 2002; Obrist et al., 2007; Pool & Geissler, 2005; Samuelsen, 2004; Smith & Mbakwem, 2007). Medically trained public and private providers (doctors and nurses) thereby account for as little as 10% of the healthcare providers in some low- and middle-income countries, the remainder comprising informal caregivers and traditional healers whose varying skills and quality can delay or undermine successful treatment (Das et al., 2008; Mwabu, 2007; Sudhinaraset et al., 2013). Complexities such as these underline the importance of understanding people's healthcare-seeking behaviour as a precondition for attaining universal health coverage and improving health in low- and middle-income countries.

The process of navigating health systems with multiple actors is captured in the concepts of “healthcare pathways” or “therapeutic itineraries:” Healthcare seeking can be understood as a multi-step process during which more than one actor or healthcare provider may be accessed. Although conceptually established and applied in qualitative research (e.g. Balabanova et al., 2009; Risso-Gill et al., 2015), the sequential understanding of healthcare-seeking behaviour has yet to permeate quantitative public health research. The majority of quantitative analyses of healthcare behaviour in low- and middle-income countries instead adopts a single-stage approach, implying that the patient “chooses” once from a portfolio of healthcare options (Gómez-Olivé et al., 2013, Hardon, 1987, Mohan et al., 2008, Moshabela et al., 2012). Such simplified single-stage analyses can help to track national progress towards achieving universal health care by providing simplified and standardised measures of healthcare access that allow for easy measurement and comparison. However, we will demonstrate in this paper that an insufficient appreciation of healthcare pathways can mislead our understanding of healthcare access.

A number of multi-purpose household surveys have started to acknowledge that healthcare-seeking involves more than one single healthcare provider (e.g. the Indian Human Development Survey, or the World Bank Living Standards Measurement Survey in Tanzania; Desai & Vanneman, 2016; National Bureau of Statistics, 2014). Yet, analyses that embrace sequential healthcare data are rare. Balabanova and McKee (2002) are a notable exception, using detailed pathway data that include up to three stages of access in order to understand patient flows through the formal health system in Bulgaria. Despite the rich and large-sample data, the pathway analysis in this study excludes informal healthcare providers and activities such as ignoring or self-treating an illness, both of which are essential for a holistic understanding of healthcare trajectories. The limited the number of reported healthcare pathways in their survey (resulting from the size of the original sample drawn, the focus on formal care, and the 4-week recall period) requires Balabanova and McKee (2002) to consolidate the pathway data into aggregate utilisation statistics for different providers, which prevents a sequence-sensitive analysis of the healthcare-seeking process. Hamid et al. (2015), who use a household survey in Bangladesh to record the first two steps of the healthcare-seeking process, apply a similarly sequence-insensitive strategy.

Whereas Balabanova and McKee (2002) and Hamid et al. (2015) analyse survey data, quantitative analyses of healthcare pathways are often based on qualitative data collection methods. For instance, Nyamongo (2002) examines the healthcare behaviour of a sample of 38 persons in Kenya over a period of ten months through ethnographic observation. The author analyses the data statistically to highlight the transitions between different forms of treatment, but the ethnographic approach is impractical for the collection and quantitative analysis of representative healthcare behaviour data.

These quantitative studies are exceptions in the literature and most studies of sequential healthcare-seeking processes are qualitative (Kibadi et al., 2009, Moshabela et al., 2011, Shaikh et al., 2008). Qualitative research is an important basis for understanding the complexity of health behaviour and for quantification through surveys (Risso-Gill et al., 2015), but the surprising absence of complementary quantitative research—which appears to result from the lack of in-depth methodological work in this area—is an obstacle to identifying representative healthcare pathways, to understanding the determinants of variations in health behaviour on the population level, and to measuring the effect of development processes and interventions on people's health behaviour. By demonstrating that the analysis of healthcare access as a sequential process can improve our understanding of people's health behaviour and its determinants, our paper aims to stimulate the more widespread use of sequential data analysis techniques in quantitative public health research.

The following section outlines the data source and survey instrument to capture healthcare-seeking sequences in our rural field sites, whereby 119 unique healthcare trajectories among 637 respondents in rural India and China emerge. Section 3 illustrates how this data can be analysed: We characterise and compare healthcare sequences in rural India and rural China (Section 3.1), demonstrate that a sequence-sensitive analysis is more precise than an aggregate analysis of healthcare access (Section 3.2), and illustrate how sequence characteristics allow for the evaluation of healthcare-seeking behaviour beyond “access / no access” indicators (Section 3.3). Section 4 summarises the limitations of this paper, compares the relative strengths and weaknesses of the sequence-sensitive and the aggregate approaches, and discusses the applicability of our method.

## 2 Material and methods^1^

**Fig. 1:**
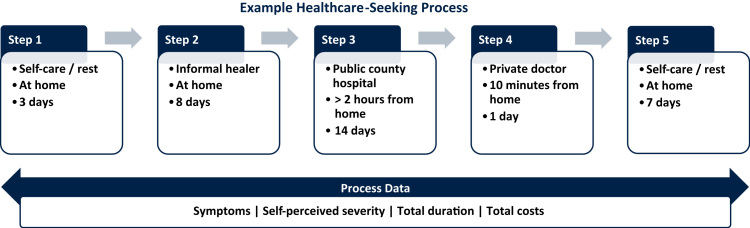
Example of healthcare-seeking process data collected in survey. Notes: example not based on actual data.

Our analysis draws on original survey data from the general population in the rural areas of Rajasthan in India and Gansu in China. The data was collected in 2014 among a cross-section of 800 adults aged 18 years and above, using a three-stage stratified cluster random sampling design. An important feature of our survey instrument was the collection of sequential healthcare pathway data that is subdivided into discrete steps of activities; their description, duration, and location; and—owing to the study focus on health-related mobile phone use—the types of mobile phone use that occurred during each step (an excerpt of the instrument is presented in the Supplementary Material, Appendix A1). In addition, we collected overarching process data including self-described symptoms and severity of the illness, the total costs incurred, and the total process duration. Fig. 1 presents a hypothetical example of such a process, where a respondent first ignores an illness after it had been detected, then engages in self-treatment with medication at home, subsequently accesses a public hospital and a private doctor, and concludes with a week-long period of continuing medication at home.

One respondent could report up to three illness episodes in our survey, including one acute “severe” illness, one acute “mild” illness, and one “long-term, recurrent, or chronic” condition. The severity of the acute illness episodes (“mild” or “severe”) was based on the respondents’ self-assessment; that is, whether *the respondent* would consider a recent illness or accident “severe” and acute (rather than e.g. recurrent or long-term). The focus on respondents’ self-assessment means that people might report an acute illness episode of an underlying yet undiagnosed chronic condition. Although this approach limits our ability to make epidemiological claims, we argue that this self- and collectively perceived severity of an illness is more relevant for people's healthcare-seeking decisions than what subsequent medical diagnosis would reveal (see e.g. Leventhal et al., 2008). We limit our analysis in this paper to acute conditions that occurred over the past year (compared to previous studies, our sample of multi-stage pathways is therefore wider but faces different recall issues – note the illustrative nature of our analysis). “Chronic and long-term” healthcare-seeking pathways follow distinctively different patterns (e.g. repeated cycles of consultation and home treatment).

Our survey captured eleven possible activities in each step and a maximum of seven steps per illness episode. The illness episode was defined by the moment from which the respondent recognised a discomfort to the point when the healthcare-seeking process stopped because the illness was cured or the symptoms simply disappeared. A healthcare-relevant activity in this survey comprised any healthcare-seeking action (advice or treatment) in relation to the illness of the respondent. Based on prior qualitative research in the same field sites, this included private and public hospitals and clinics, pharmacies, shops selling medicine, traditional healers, providers of alternative medicine, self-care through resting or medication at home, help from family and friends, an “other” option, and whether the responded simply ignored the condition (particularly common at the beginning of an illness).

**Fig. 2:**
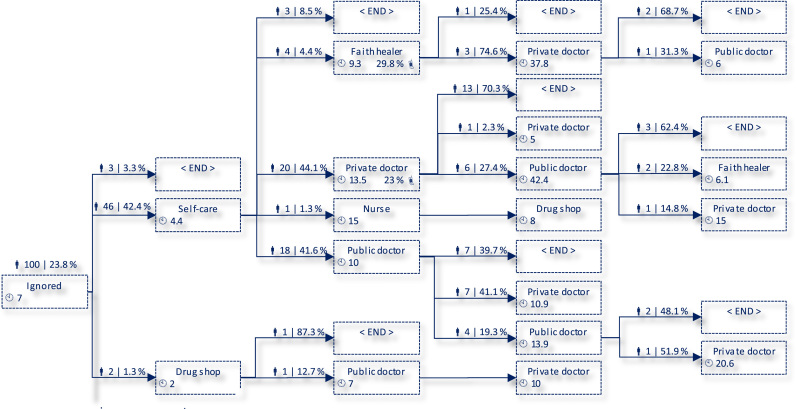
Excerpt of Healthcare-Seeking Processes in Rajasthan (Mild Illness). Notes:  denotes number of observations and weighted share of population at each step (using census data).  denotes population-weighted average duration of step in days.  denotes population-weighted portion of people in each step using a mobile phone in relation to the illness.

Source: Own illustration, derived from fieldwork data.

The pathway data resulted in 119 unique sequences among 637 respondents in India and China who reported at least one “mild” or “severe” illness. An excerpt of these healthcare sequences representing 15% of the reported mild illnesses in Rajasthan is shown in Fig. 2. In the remainder of this paper, we will reduce the spectrum of possible activities for simplicity to “no care or self-care” (e.g. including self-treatment with medicines stored at home), “care from informal providers, family, and friends” (e.g. including faith healers, family members, and kiosks selling drugs), “private healthcare” (i.e. private clinics and hospitals), and “public healthcare” (e.g. village-level doctors and nurses, community hospitals). Despite the reduction of dimensions, 80 unique sequences among the 637 respondents remain.

Our analysis compares three types of sequential analysis to conventional aggregate analysis of healthcare access. During this analysis, we will make assumptions about how to express “representativeness” of healthcare-seeking processes, about how healthcare-seeking behaviour might be influenced by mobile phone use, and about which kinds of behaviours align with hypothetical “ideal” processes in light of people's self-described symptoms. We make these assumptions and analytical choices for the purposes of illustrating the application and usefulness of quantitative methods to healthcare-seeking processes, acknowledging that they are debatable. We will explain our modelling choices in the respective parts of the analysis section of this paper.

In the first part of our analysis, we characterise and compare the healthcare behaviour in the two field sites using descriptive analysis. Whereas conventional (i.e. aggregate) descriptive statistics of healthcare access focus on health provider utilisation rates, sequential data enables insights into the structure of common pathways to care and their attributes (e.g. average time spent in each step of the healthcare-seeking process).

Secondly, we assess the influence of technology on healthcare-seeking process attributes—in this case, the effect of health-related mobile phone use on the delay of accessing specific healthcare providers. A sequence-insensitive approach would juxtapose phone ownership/use and healthcare access, assuming that they overlap. In contrast, a sequence-sensitive analysis assesses whether the event occurred during the relevant steps of the process, making it in theory a more precise methodology. We compare the estimated delays of healthcare access for the sequence-sensitive and -insensitive approach in order to demonstrate the superiority of the former over the latter.

Thirdly, we highlight the difference between sequence-sensitive and -insensitive evaluations of healthcare access—that is, whether a healthcare-seeking process complies with illustrative “ideal” processes of healthcare access. This normative dimension is often only implicit in sequence-insensitive analyses that assess whether any kind of healthcare provider was accessed during an illness (Herbert et al., 2012). Sequence-sensitive designs enable explicit and more refined appraisals of the nature of the process, for example whether an informal provider was accessed before a formal one, or whether referral mechanisms were bypassed in the process. For illustrative purposes, we evaluate people's healthcare-seeking processes as a function of their household wealth.

Our analyses were carried out with Stata and R's “TraMineR” package (Gabadinho et al., 2011a, Gabadinho et al., 2011b, StataCorp, 2013). Stata also offers the user-written “SADI” package for the sequence analysis methods outlined here (Halpin, 2014).

## 3 Analysing sequential healthcare behaviour data

### 3.1 Descriptive analysis

The first part of our analysis will explore healthcare-seeking behaviour descriptively, comparing conventional and sequential measures. In contrast to aggregate healthcare access statistics, sequence analysis offers a range of tools to describe, group, and analyse healthcare pathways as a whole or parts thereof.

**Fig. 3:**
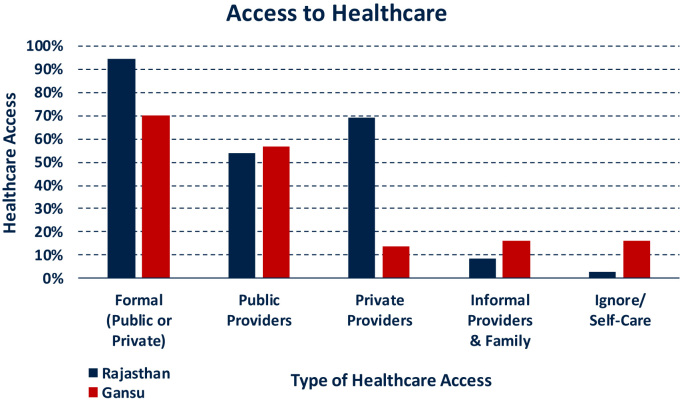
Aggregate Access to Healthcare. Notes: Reporting on illness episode level. Respondents can have access to various providers during the same healthcare process. Statistics are population weighted across the field site districts using census data and according to number of mild and severe illnesses per person. Proportions as share of patients reporting a mild or severe illness.

In the absence of sequential data, descriptive analyses of healthcare seeking are normally limited to the utilisation rates of various providers. For instance, Fig. 3 below indicates that overall access to formal (public and private) providers is higher in rural Rajasthan, and that private healthcare plays a larger role in Rajasthan than it does in Gansu, where public healthcare providers dominate. The wide availability of medication among Gansu households and practitioners that we observed in our preceding qualitative work (Haenssgen, 2014) may explain the relatively large share of people not accessing any healthcare provider during an illness. Overall, such descriptions can be informative as they permit a swift (albeit superficial) characterisation of the sites in terms of healthcare utilisation rates.

**Fig. 4:**
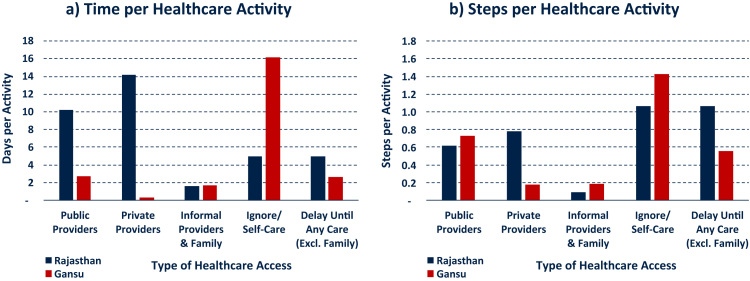
Aggregate characteristics of healthcare-seeking process. Notes: Reporting on illness episode level. Statistics are population weighted across the field site districts using census data and according to number of mild and severe illnesses per person. Time reported in days.

Where sequential data has been collected, further descriptive statistics can for example report the average time and steps devoted to each healthcare provider during an illness (this approach still disregards the sequence of events). Comparing such statistics provides additional information on the healthcare-seeking behaviour of rural dwellers in Rajasthan and Gansu: As can be seen in Fig. 4, Panel a, patients in Rajasthan spend most of their illness time in treatment with private and public doctors, whereas self-care and no care at all are most extensive health-related activities in Gansu. Patients in Gansu are also faster in accessing healthcare by an average of 2.4 days. Panel b of Fig. 4 provides similar information, yet with a focus on the number of healthcare activities involved in each illness episode. Among others, this graph shows that patients in rural Rajasthan do not only spend more time before accessing healthcare providers, but also engage in more activities beforehand.

**Fig. 5:**
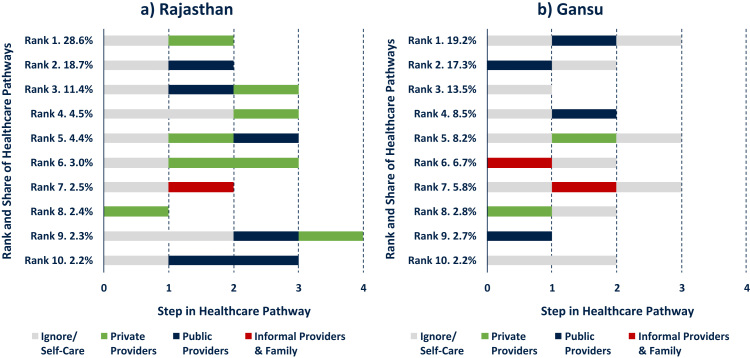
Ten Most Common Healthcare Pathways. Notes: Ranks and shares of pathways are population weighted across the field site districts using census data and according to number of mild and severe illnesses per person. Panel a accounts for 80.0%, Panel b for 86.9% of all “mild” and “severe” healthcare pathways in Rajasthan and Gansu, respectively.

In order to acknowledge the sequential nature of healthcare-seeking behaviour explicitly, it is possible to consider common and representative pathways across the two field sites. For instance, Fig. 5 displays the ten most common healthcare trajectories and their respective share of all pathways in each country sample; Panel a for Rajasthan and Panel b for Gansu. This ranking was established by counting the occurrence of the various unique pathways in the data, weighted by the population that each respondent represented, and reported as a share of all illness episodes (this process can be automated in software packages like TraMineR in R, or done manually by sorting and counting the pathway data, e.g. an ascending sort by Step 1, Step 2, etc.). The graph illustrates that the majority of illness episodes in both field sites begin with a “wait and see” phase and often involve combinations of different healthcare providers. (Further analysis could differentiate illness episodes by duration [e.g. 3 days spent ignoring the illness, 2 days in the care of a public provider, etc.] rather than only by their number of steps.).

**Fig. 6:**
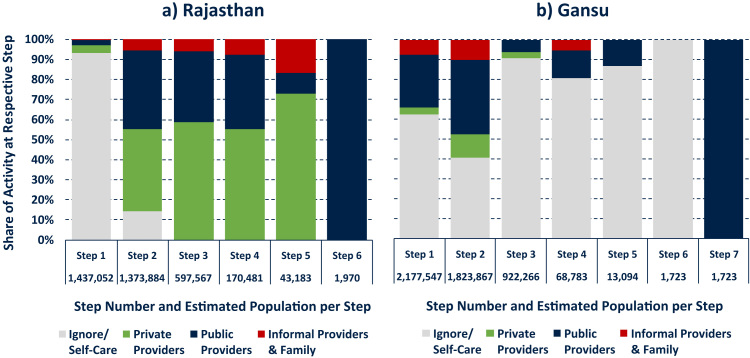
Distribution of activities at each “Step” of Healthcare Pathway. Notes: statistics weighted using census data and according to number of reported illnesses.

In order to handle the arguably complex sequence data more effectively, it can be helpful to characterise the overall distribution of healthcare-seeking activities. For example, Fig. 6 plots the distribution of activities for each individual step in the healthcare-seeking process in both field sites (note that later stages of the process have a smaller sample size). The graphs indicate that it is more common to not involve third parties in personal healthcare in the Gansu field site throughout the healthcare-seeking process. At the same time, Gansu respondents are more likely than their Rajasthan counterparts to access public healthcare as soon as an illness is detected. The Rajasthan data further indicates that longer healthcare pathways are associated with an intensification of private healthcare utilisation.

While numerous more applications of the exploratory sequence analysis are conceivable,^2^ this outline illustrates its potential to complement conventional descriptive analyses of healthcare-seeking behaviour. Conventional aggregate statistics enable a first overview of healthcare access and utilisation patterns among different groups and populations (Corno, 2014; Herbert et al., 2012; Srivastava & McGuire, 2015). Sequential data and sequence analysis offer further insights into the characteristics of common healthcare processes, for example the time spent in various healthcare activities, common healthcare pathways across different groups, or shifts in healthcare provider access during the course of an illness. Such additional information can help to establish a more encompassing, informative, and faithful representation of people's healthcare-seeking behaviour.

## 3.2 Sequence-sensitive determinants of pathway attributes

Sequential data does not only permit more comprehensive descriptive analysis, but it also enables more precise estimation in statistical analyses that explore the determinants of healthcare-seeking process characteristics. We exemplify the value of sequence-sensitive estimators through the case of health-related mobile phone use and its association with delays to healthcare access.

**Fig. 7:**
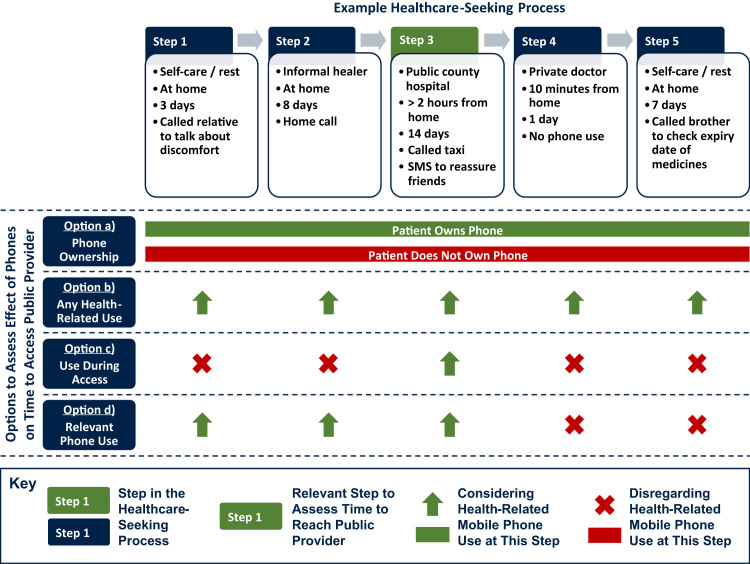
Four options to assess the effect of phone use on time to access public healthcare.

The effect of mobile phone use (or any other health-relevant activity) during the healthcare-seeking process can be assessed in various ways, and Fig. 7 presents four options, based on a process where we are interested in the time until a patient reaches a public hospital in Step 3. Three plausible, sequence-insensitive approaches assess healthcare access depending on whether the patient owns a mobile phone (Option a in Fig. 7), whether any health-related mobile phone use took place during the illness (Option b), or whether the patient used a mobile phone in order to access the public hospital (Option c). All these approximations have clear limitations: Option a might not involve any health-related use at all even if the patient owns a phone, Option b potentially includes irrelevant mobile phone uses *after* the public hospital was accessed, and Option c disregards the cumulative nature of delays prior to healthcare access in Step 3 (e.g. if the patient talked about her or his illness to a family member in Step 1). We argue that, if there is any relationship between healthcare access and phone use, it should be in relation to phone use that occurred *before or when* the provider in question was accessed. Rather than approximating, sequence-sensitive data permits us to measure the relevant health-related mobile phone use directly and thus more precisely, displayed as Option d in Fig. 7.

To illustrate the imprecision of a sequence-insensitive approximation of health-related phone use, we regress delays in healthcare access against the four options to assess health-related mobile phone use and compare the estimated predicted changes of such phone use to the sequence-sensitive Option d. Table 1 presents these predictions (detailed results of the 16 estimated models are presented in the Supplementary Material, Appendix A2), based on negative binomial regression models with village-cluster-robust standard errors. The regression model is expressed as

$$P\left(y_{i}|x_{i},\alpha \right)=\left(\left[\Gamma (y_{i}+\alpha^{–1})\right]/\left[\Gamma \left(y_{i}+1\right)\Gamma (\alpha^{–1})\right]\right){\left[\alpha^{–1}/(\alpha^{–1}+\mu_{i})\right]}^{\alpha -1}{\left[\mu_{i}/(\alpha^{–1}+\mu_{i})\right]}^{yi},$$

where Γ(·) is the gamma function, α is the dispersion parameter, *y*_*i*_ is the delay to healthcare access during illness episode *i*, and ln(μ_*i*_)=**β*****x***_*i*_ is the natural log of the mean (Cameron & Trivedi, 1998; Rabe-Hesketh & Skrondal, 2012). The delays are calculated for four different types of access, namely (1) any formal or informal provider (excluding family/friends), (2) public healthcare access, (3) private, and (4) informal providers (including family/friends). The vector of covariates **β*****x***_*i*_ contains health-related mobile phone use during the illness episode *i* (measured using each of the four options separately) in addition to control variables for common determinants of healthcare access, including self-reported disease severity, individual characteristics (sex, literacy, education, age, health status and ability to carry out activities of daily living), household characteristics (size, wealth, mass media and vehicle ownership, sex and education of household head, mobility patterns of family members), knowledge of phone-based health services (hotlines and ambulances), health provider preferences, health system characteristics (distance to nearest doctor, time until ambulance would arrive in village), and country dummies (on determinants of healthcare seeking, see e.g. Kroeger, 1983; Nyamongo, 2002; Shaikh et al., 2008). We use negative binomial regression models because the delay data is over-dispersed (i.e. the variance of the dependent variable exceeds its mean; Johnson, 2004). Multilevel specifications of these models (modelling disease episodes as nested in individuals, who are in turn nested in villages) were inferior to single-level estimations. Interaction models (including interaction terms between country dummy and health-related mobile phone use) proved insignificant with one exception (access to public providers and phone use during access as in Option c) and are therefore omitted from the reporting.

**Table 1:** Average predicted absolute increase in healthcare access delay for phone users (in days).

	**Sequence-insensitive measures**						**Sequence-sensitive measure**		**Difference between delay estimates based on sequence-insensitive and sequence-sensitive measures in percent**					
	**Option a) Phone ownership**		**Option b) Any health- related use**		**Option c) Use during access**		**Option d) Relevant phone use**							
	**Rajasthan**	**Gansu**	**Rajasthan**	**Gansu**	**Rajasthan**	**Gansu**	**Rajasthan**	**Gansu**	**Rajasthan**			**Gansu**		
	**(1)**	**(2)**	**(3)**	**(4)**	**(5)**	**(6)**	**(7)**	**(8)**	**(9) [(1) - (7)] (7)**	**(10) [(3) - (7)] (7)**	**(11) [(5) - (7)] (7)**	**(12) [(2) - (8)] (8)**	**(13) [(4) - (8)] (8)**	**(14) [(6) - (8)] (8)**
**Any Healthcare Provider (*****n*****=592)**	0.0^a^	0.0^a^	+11.0	+3.0	+6.0	+1.9	+7.4	+2.1	–100%	+50%	–18%	–100%	+42%	–12%
**Public Providers (*****n*****=380)**	0.0^a^	0.0^a^	+34.2	+3.6	+39.2	+3.7	+41.8	+4.0	–100%	–18%	–6%	–100%	–9%	–5%
**Private Providers (*****n*****=264)**	0.0^a^	0.0^a^	+10.5	+1.1	0.0^a^	0.0^a^	+7.3	+0.7	–100%	+43%	–100%	–100%	+43%	–100%
**Informal Providers (*****n*****=81)**	+63.8	+0.9	+168.5	+1.3	0.0^a^	0.0^a^	0.0^a^	0.0^a^	N/A	N/A	N/A	N/A	N/A	N/A

*Notes:* Predicted values based on negative binomial regression model with village-cluster robust standard errors. Observations at the illness episode level, with up to one self-reported “mild” and “severe” illness per person.

a Coefficient in negative binomial regression model was not statistically significant at the 10% level.

The results in Table 1 show the estimated change of delays to healthcare as a function of health-related phone use, holding all other variables constant at sample means (for a discussion as to why mobile phone use would be associated with increased delays in healthcare access, see Haenssgen, 2015). Coefficients insignificantly different from zero at the 10% level are reported as zero predicted change. The table illustrates that sequence-insensitive measures of health-related phone use (Columns 1 to 6) deviate notably from the sequence-sensitive approach (Columns 7 and 8). The relative difference of the marginal effects of the sequence-insensitive vs. the sequence-sensitive measures is shown in the latter part of the table (Columns 9 to 14). The sequence-insensitive models overstate the delay in access by as much as 50% in the case of access to any healthcare providers (any health-related phone use; Column 10) and understate them by 100% where the regression coefficients were statistically insignificant (e.g. phone use when accessing private providers; Columns 11 and 14). Moreover, in particular the regression models based on mobile phone ownership (Option a; Columns 1 and 2) yield statistically significant results where there are plausibly none (informal access, compare Columns 7 and 8), or are statistically insignificant where the more precise estimators indicate an association at the 1% level (see Appendix Table A2 in the Supplementary Material for detailed results). Similarly concerning patterns can be observed for models based on Options b and c. If we were to rely on common yet sequence-insensitive proxy indicators of health-related mobile phone use, we would for instance incorrectly detect a statistical relationship between phone use and informal healthcare access because phones are more commonly used *after* people accessed informal providers.

The wide and inconsistent variation of results using sequence-insensitive indicators in this example shows that sequence-sensitive measures of technology use can offer superior analytical value for assessments of delays in healthcare access. Sequence-insensitive measures would have vastly over- or understated the predicted outcomes in some cases, and in other cases would have found statistically significant relationships where there are possibly none.

## 3.3 Healthcare pathway evaluations

This last sub-section describes how sequential data enables more encompassing evaluations of people's healthcare pathways. Rather than merely assessing whether particular types of healthcare were accessed (which is implicitly normative), it is for instance possible to make explicit judgements whether patients adhered to referral procedures that are intended to improve health system efficiency, or whether patients delayed public or private treatment by first visiting untrained informal healers.

**Fig. 8:**
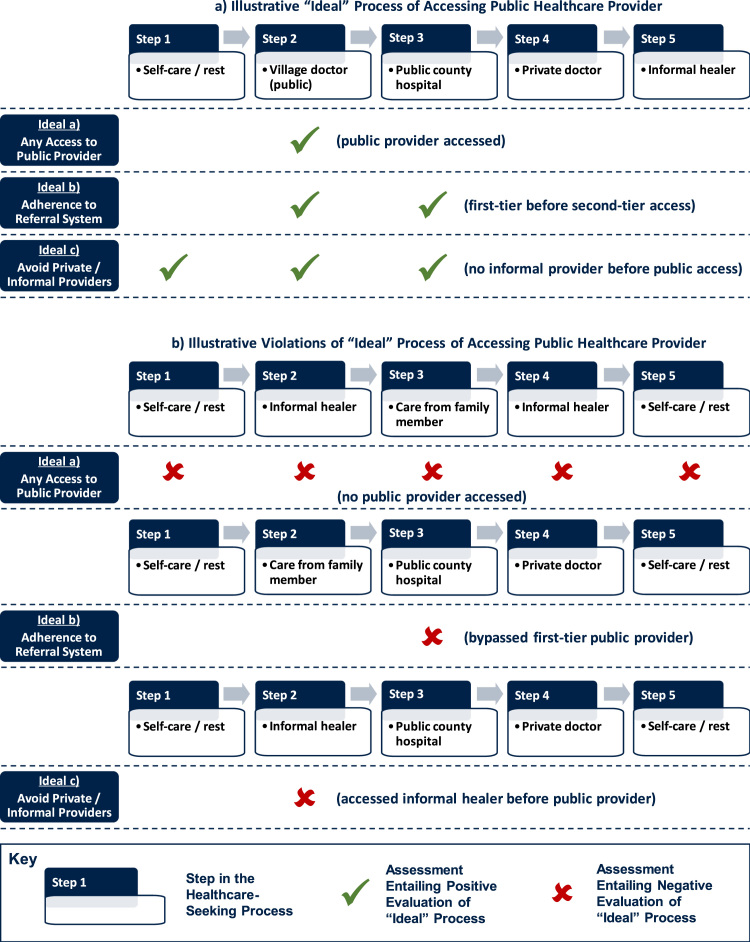
Examples of “Ideal” pathways and violations where patient ought to access public care.

In our example, we will make these evaluations as assessments against hypothetical “ideal” behaviours. Our illustrative definition of “ideal” behaviour involves access to regulated healthcare providers and minimal delays to care where the illness requires the patient to do so. The assessment criteria of what counts as “ideal” are externally imposed on grounds of health system efficiency and concerns about delays in treatment (explained below and exemplified in Fig. 8). This does not necessarily mean that patients or healthcare providers adhere to the same criteria when they make their choices, and their personal notions of an “ideal” process can very plausibly differ (and for good reasons, including e.g. health knowledge, societal perceptions of the health condition, concerns about provider competence, or variations in decision-making autonomy; see previously cited references on healthcare-seeking determinants). Therefore, we do not judge whether a patient is right or wrong in their personal choices, but we make a judgement as to whether patient behaviour aligns with the externally imposed evaluative criteria of the analyst, which may pertain e.g. to health system efficiency.

We make our evaluations with reference to public healthcare access in rural Gansu and Rajasthan, considering that our field sites are particularly resource-constrained (people in remote villages often have to walk for half an hour to reach a clinic), and the health system in rural Rajasthan is particularly fragmented with a broad range of limitedly regulated private and informal healthcare providers. We exemplify the application of sequence-sensitive evaluations by examining if wealthier households are more likely behave in line with (illustrative) “ideal” processes of healthcare utilisation, immediacy of treatment from public healthcare, and adherence to referral procedures. Other researchers may want to articulate specific hypotheses about the determinants of healthcare-seeking, examine behaviours for different health issues with fundamentally different pathway profiles (e.g. communicable vs. non-communicable diseases, childbirth), or make different assumptions as to what constitutes ideal healthcare-seeking processes depending on their study focus and the local health system context.^3^ Our methods allow such a flexible and transparent adaptation of the analysis.

Fig. 8 exemplifies our three illustrative approaches to evaluating “ideal” healthcare pathways, assuming that a patient should get treatment from a public healthcare provider (see below on how we judge whether medical care is required). Panel a displays hypothetical “ideal” pathways to public healthcare; Panel b contains examples where such an “ideal” pathway is not adhered to. The evaluation of hypothetical “Ideal a” follows the conventional, sequence-insensitive approach and considers only whether any public healthcare access took place during an illness, leading to a negative evaluation where this is not the case (see Panel b). The evaluation of “Ideal b” considers the process of accessing public providers, with a negative evaluation if referral systems are bypassed. Whereas the “ideal” process in Panel a involved a visit to a local doctor prior to public hospital access, the “ideal” is violated if did not contain such a first-tier provider visit. Lastly, from a health system utilisation perspective, analysts might also be interested whether patients delayed their treatment by approaching untrained, unregulated, or costly private and informal practitioners prior to visiting public healthcare providers. The evaluation of “Ideal c” in Panel a demonstrates accordingly how a healthcare pathway might comply with this “ideal” if no such contact took place *before* the public doctors and hospitals were accessed, in contrast to the violation of the hypothetical “ideal” in Panel b. Such process benchmarks are clearly normative and context-specific, but they can be tailored and specified transparently if sequential healthcare-seeking data is available, thus going beyond binary measures of “access” or “no access.”

In our present example, we explain the people's compliance with hypothetical “ideal” processes of public healthcare utilisation as a function of their household wealth (and other determinants of healthcare seeking as in Section 3.2), hypothesising for illustrative purposes that household wealth is associated positively with higher degrees of compliance. We determine whether an ill patient should seek medical treatment based on their self-described symptoms, applying relatively strict decisions whether they should see a public doctor in the particularly resource-constrained health systems of rural Rajasthan and rural Gansu (e.g. in the case of jaundice or a leg fracture, but not for headaches or fever; this illustration could be relevant for healthcare resource allocation in a particularly resource-scarce environment, but other analyses and contexts could flexibly apply different criteria). Public health specialist Dr. Proochista Ariana validated the disease classification (22 categories) and the *ex post* assessment of medical need. We assess household wealth based on an asset and amenity index, established through principal component analysis and sub-divided into population-weighted quintiles separately for each country. We estimate the relationship through a logistic regression model, given the binary nature of compliance with “ideal” processes as the dependent variable. Corresponding to the evaluation approaches for the “ideal” processes a, b, and c above, our models consider access to public healthcare (a) without restrictions, (b) that follows referral systems for self-described “mild” cases (i.e. we permit that this may be ignored in emergencies), and (c) that discourages private and informal healthcare providers (other than family and friends) prior to accessing public facilities.

The basic logistic regression model is specified as logit[P(*y*=1 | ***x***_*i*_)]=**β*****x***_*i*_, with the probability of success P(*y*=1) expressed as the natural log of the odds of achieving a positive result, conditional on a vector of covariates ***x***_*i*_ (Hilbe, 2009). ***x***_*i*_ contains the same 29 determinants of healthcare access as described in Section 3.2 (except health-related mobile phone use, which is not a focal variable in this illustration). Three-level random intercept multilevel specifications did not improve model fitness compared to single-level logit models. Interaction terms allowing the slope of the wealth index quintile coefficient to vary across the field sites were statistically insignificant; interaction models are therefore omitted from the presentation.

Based on our model, the hypothesis of a positive relationship between wealth and “ideal” public healthcare utilisation would be supported if the coefficient of the wealth index variable is positive and statistically significant (using a 10% significance level as threshold). The main results are shown in Table 2, omitting the 28 other control variables (detailed results are presented in the Supplementary Material, Appendix A3). The principal insight of the table is that the overarching, sequence-insensitive evaluation of public healthcare access appears to be independent of household wealth (Column 1; this controls for other determinants of healthcare access). While this suggests that wealthier households utilise public healthcare in similar ways as poor households, the sequence-sensitive evaluations for “Ideal b” and “Ideal c” in Columns 2 and 3 are statistically significant at the 5% level. The seemingly more widespread consultation of private and informal providers prior to visiting public doctors (Column 3) corresponds to higher affordability of out-of-pocket payments for more affluent groups. However, as household wealth is associated with bypassing referral systems (Column 2), poorer households may be crowded out at higher tiers of the public healthcare system.

**Table 2:** Main results of logit regression models to evaluate “ideal” processes of public healthcare access.

	**Sequence insensitive**	**Sequence sensitive**	
	**Evaluation of “Ideal a)”**	**Evaluation of “Ideal b)”**	**Evaluation of “Ideal c)”**
	**Any public healthcare access**	**Public healthcare access with adherence to referral**	**Public healthcare access Without prior informal/Private care**
	**Coefficient (Standard error)**	**Coefficient (Standard error)**	**Coefficient (Standard error)**
	**(1)**	**(2)**	**(3)**
**Household Asset Index Quintile**	−0.135 (0.082)	−0.258^**^ (0.110)	−0.228^**^ (0.102)
**Pseudo R**^**2**^	0.093	0.320	0.270
**Number of Observations**	669	649^a^	669

*Notes:* Coefficients reported. Standard errors in parentheses. 28 control variables and constant omitted.

*p < 0.1, **p < 0.05, ***p < 0.01.

a Variable “preference for other health providers” predicts failure perfectly. Variable dropped and 20 observations not used.

While all models reject the hypothesis that wealthier households exhibit a higher degree of compliance with hypothetically “ideal” healthcare behaviour, the sequence-insensitive evaluation would have suggested that no difference between more and less affluent individuals exists. In contrast, sequence-sensitive evaluations hint at potentially detrimental behaviours of wealthier households, especially from a health system resource allocation perspective.

We could of course debate whether self-reported symptoms are reliable, whether our diagnosis of healthcare-worthy illnesses is sound, and whether our process benchmarks are appropriate. The fact that these points are debatable reflects on their explicit, transparent, and flexible nature. In the absence of sequential data, evaluations of people's health behaviour would be limited to the “any access” scenario for specific diseases. This would not have permitted us to detect the potentially problematic nature of household wealth in our example.

## 4 Discussion

This paper illustrated the use of analytical tools to capture and assess sequential healthcare behaviours. We demonstrated three possible applications, using primary survey data from two rural low- and middle-income contexts. The first application involved the descriptive analysis of healthcare sequences in order to characterise and contrast the behavioural patterns of rural dwellers in Rajasthan and Gansu beyond simplistic healthcare utilisation statistics. The second illustration demonstrated the value of sequence-sensitive indicators when modelling the influence of technology use (here: mobile phones) on healthcare-seeking process outcomes like the delays to access public health workers. Among others, the analysis showed that conventional or sequence-insensitive proxy measures overstate effects by as much as 50 percent, or detect statistical relationships where none might exist. Lastly, we exploited the sequential structure of our data to develop a new evaluation of health system utilisation, both from a health system (referral procedures) and from a service delivery perspective (informal and private care before public healthcare access). In the context of public healthcare access as a function of people's household wealth, an analysis that focuses on overall utilisation rates finds no statistical association with household wealth, whereas sequence-sensitive assessment criteria detect potentially problematic patterns of referral system bypassing and more extensive health system utilisation prior to accessing public healthcare among wealthier households.

Sequential healthcare behaviour data and its analysis are clearly not without challenges. The first major limitation of our approach is that actual healthcare behaviours need not necessarily follow a linear sequence of discrete steps, but might rather involve parallel and overlapping activities, not all of which can be clearly defined as “healthcare seeking.” Future methodological work may thus develop our quantitative measures further in order to represent complex health behaviours more realistically. Secondly, the retrospective assessments of healthcare pathways through survey data can create problems if the recall period is too long, and our methodological individualism excludes individuals who did not survive an illness. Prospective studies may be desirable in theory to overcome these problems, but they come with their own set of logistical and methodological challenges (Miller & Salkind, 2002). Lastly, analyses and normative evaluations of healthcare sequences require a complementary understanding of the supply-side quality and capacity of healthcare providers, their actual practices, social norms and preferences, and, ideally, treatment outcomes in order to be truly informative and sensitive to local contexts. However, it is worth reiterating that these limitations are not unique to sequence-sensitive analysis alone, as they apply to conventional measures of healthcare access just as well.

Considering the juxtaposition of sequence-sensitive and -insensitive methods in this paper, it is clear that both have their strengths and weaknesses. Compared to sequence-sensitive approaches, conventional data and sequence-insensitive methods lead to a radical simplification of people's actual behaviours that can obscure interesting and important healthcare-seeking patterns. But they enable straightforward descriptions of healthcare utilisation rates, they can serve as proxy variables of health behaviour to a limited extent, and, perhaps most importantly, they can be extracted easily from many existing data sets. Especially the latter point renders sequence-sensitive approaches often infeasible, however superior they may be. Yet, where sequential data generation is possible, it can facilitate and improve (a) the description of behavioural landscapes in order to guide health policy and educational activities, (b) the detection of (problematic) behavioural patterns and their underlying drivers, and (c) the understanding of the association between technology or other healthcare solutions (e.g. vehicles) and people's healthcare decisions (note that further methodological work is required to establish causal relationships).

In conclusion, our methodological contribution to the quantitative study of health behaviour was motivated by the gap between the conceptually established understanding of healthcare-seeking as a sequential process on the one hand, and the lack of transparent and rigorous methodology to assess such sequences statistically on the other hand. By providing a survey instrument and analysis techniques for sequential healthcare data, we hope to nurture a more comprehensive, detailed, and precise understanding of people's health behaviour, considering the persistent burden of premature death in low- and middle-income countries.

## Footnotes

1 The research was approved by the Social Sciences and Humanities Inter-Divisional Research Ethics Committee of the University of Oxford, by the Gansu Province Department of Statistics (Ref. 2014/8), and by the internal ethics commission of the Indian Institute of Health Management Research, Jaipur.

2 For example, cluster analysis can help to establish representative healthcare sequences to characterise different populations (Gabadinho et al., 2011a). It also enables multivariate analysis to identify factors that explain the differences among the observed sequences (Studer et al., 2011). Event history analysis and latent class growth modelling can be applied to sequential data as well (Abbott, 1995:104-105; Eerola & Helske, 2012; Gabadinho et al., 2011a).

3 Note that we are not comparing pathways per se as dependent variable, but the evaluation outcomes of those pathways. This permits us to subsume a broad range of behaviours, all of which can receive a bespoke assessment as to whether they are in line with ideal pathways to healthcare. Different disease categories can then for instance be analysed as independent control variables.
