# Supplementary material for: Healthcare access: A sequence-sensitive approach
Source: SSM Popul Health. 2016 Nov 30;3:37–47. doi: 10.1016/j.ssmph.2016.11.008 (PMC5769022; doi:10.1016/j.ssmph.2016.11.008)
Supplement: Supplementary file 1 — Supplementary material [file mmc1.pdf]

Appendix A1. Excerpt of Survey Instrument to Capture Sequential Healthcare Pathways (Severe Illness)

24. Did you have **what you would consider** a severe acute illness (not a chronic, long-term condition that comes again and again) or a severe accident in the last twelve months?<br><br>[***“severe” according to the perception of the respondent***]<br>[***if disease more than 52 weeks / one year, this will be response for chronic disease. Ask for any other severe illness.***]<br>[***if no, continue with Question 25***]	No ....2 ➔ [***go to Q 25***]<br>Yes.........1 ↓
[***if yes***:]<br>24.1. Can you please describe the symptoms or problem? Please also state if you received a diagnosis.	Description of condition:<br>______________________________________<br>______________________________________<br>______________________________________
24.2. Was it an emergency situation?	Yes.........1<br>No..........2
24.3. How long did the illness last, from the moment you detected it to the moment it was cured (or to today otherwise)?	Duration: ______ days / ______ weeks / _____ months
24.4. How much money did you spend on the treatment in total? This includes all costs that are linked to the condition, for example transportation, fees, costs of medicine, and so forth.<br><br>[***if respondent very unsure, ask: “was is rather 100 Yuan, 1,000 Yuan, or 10,000 Yuan?”***]	Total cost of treatment: RMB __________________

24.5. Can you please explain the stages of the treatment? I will ask you step-by-step what you did, starting from the moment you first detected the condition.
[*ask respondent what he or she did first, then code answer and continue. After each row, ask: "What did you do next?" Only one activity per step. If e.g. medical treatment and then home care, first step is medical treatment, second step is home care. If [next step], cross out the remainder of the row. Repeat until respondent was cured.*]

Step	a) What kind of help or treatment did you get at this stage? [*if unsure, please specify*]	b) Where did this activity take place?	c) How long did this stage last? [*let respondent choose category; if less than one day, code "1" day*]	d) Was a mobile phone used during this stage in connection with your condition? [*if no, next step*]	e) What was the purpose of using the mobile phone? [*Mark all that apply*]	f) Who was contacted? [*Mark all that apply*]	g) Who used the mobile phone? [*Mark all that apply*]	h) How was the phone used? [*Mark all that apply*]
	Ignored /did nothing ....1<br>Self-care (sleep, rest, medication at home) ....2<br>Care from family and friends (full-time)....3<br>Treated / consulted by a pharmacist ....4<br>Treated / consulted by someone else who sells drugs ....5<br>Treated / consulted by priv. doctor/hospital ....6<br>Treated / consulted by village doctor ....7<br>Treated / consulted by a gvt. doctor/hospital ....8<br>Other (specify) 9	At home....1<br>Less than 10 minutes from home ....2<br>10 to 29 minutes ....3<br>30 to 59 minutes ....4<br>60 to 119 minutes ....5<br>2 hours or more from home 6		Yes ....1<br>No 2	Ask for advice....1<br>Call for help....2<br>Arrange transport ....3<br>Appointment....4<br>Reassure family / friends ..5<br>Ask for money / supplies ..6<br>Provider contacting me for information ....7<br>Reminder for treatment ...8<br>Other (specify) 9	Nobody ....1<br>HH member ....2<br>Family member outside HH ....3<br>Other relative....4<br>Neighbour ....5<br>Friend other than neighbour ....6<br>Other villager ....7<br>Medical provider.8<br>Ambulance....9<br>Other....10	Myself....1<br>Spouse ....2<br>Parents ....3<br>Children ....4<br>Siblings....5<br>Other family member / relative....6<br>Neighbour....7<br>Friend other than neighbour ....8<br>Other villager ....9<br>Other 10	Call ....1<br>SMS ....2<br>Internet, messenger....3<br>Alarm etc.....4<br>Other (spec.) 5
24.5.1. Step 1 (detection)	1 2 3 4 5 6 7 8<br>9: ______	1 2 3 4 5 6	Days: ___<br>Weeks: ___<br>Months: __	Yes ....1 ➔<br>No .....2 🡻<br>[*next step*]	1 2 3 4 5 6 7 8<br>9: ______	1 2 3 4<br>5 6 7 8<br>9 10	1 2 3 4<br>5 6 7 8<br>9 10	1 2 3 4<br>5: ______
24.5.2. Step 2	1 2 3 4 5 6 7 8<br>9: ______	1 2 3 4 5 6	Days: ___<br>Weeks: ___<br>Months: __	Yes ....1 ➔<br>No .....2 🡻<br>[*next step*]	1 2 3 4 5 6 7 8<br>9: ______	1 2 3 4<br>5 6 7 8<br>9 10	1 2 3 4<br>5 6 7 8<br>9 10	1 2 3 4<br>5: ______
24.5.3. Step 3	1 2 3 4 5 6 7 8<br>9: ______	1 2 3 4 5 6	Days: ___<br>Weeks: ___<br>Months: __	Yes ....1 ➔<br>No .....2 🡻<br>[*next step*]	1 2 3 4 5 6 7 8<br>9: ______	1 2 3 4<br>5 6 7 8<br>9 10	1 2 3 4<br>5 6 7 8<br>9 10	1 2 3 4<br>5: ______
24.5.4. Step 4	1 2 3 4 5 6 7 8<br>9: ______	1 2 3 4 5 6	Days: ___<br>Weeks: ___<br>Months: __	Yes ....1 ➔<br>No .....2 🡻<br>[*next step*]	1 2 3 4 5 6 7 8<br>9: ______	1 2 3 4<br>5 6 7 8<br>9 10	1 2 3 4<br>5 6 7 8<br>9 10	1 2 3 4<br>5: ______
24.5.5. Step 5	1 2 3 4 5 6 7 8<br>9: ______	1 2 3 4 5 6	Days: ___<br>Weeks: ___<br>Months: __	Yes ....1 ➔<br>No .....2 🡻<br>[*next step*]	1 2 3 4 5 6 7 8<br>9: ______	1 2 3 4<br>5 6 7 8<br>9 10	1 2 3 4<br>5 6 7 8<br>9 10	1 2 3 4<br>5: ______
24.5.6. Step 6	1 2 3 4 5 6 7 8<br>9: ______	1 2 3 4 5 6	Days: ___<br>Weeks: ___<br>Months: __	Yes ....1 ➔<br>No .....2 🡻<br>[*next step*]	1 2 3 4 5 6 7 8<br>9: ______	1 2 3 4<br>5 6 7 8<br>9 10	1 2 3 4<br>5 6 7 8<br>9 10	1 2 3 4<br>5: ______
24.5.7. Step 7	1 2 3 4 5 6 7 8<br>9: ______	1 2 3 4 5 6	Days: ___<br>Weeks: ___<br>Months: __	Yes ....1 ➔<br>No .....2 🡻<br>[*next step*]	1 2 3 4 5 6 7 8<br>9: ______	1 2 3 4<br>5 6 7 8<br>9 10	1 2 3 4<br>5 6 7 8<br>9 10	1 2 3 4<br>5: ______

Appendix A2. Full Results of Negative Binomial Regression Models for Delays to Healthcare Access

	Sequence-Insensitive Measures												Sequence-Sensitive Measure			
	Option a) Phone Ownership				Option b) Any Health-Related Use				Option c) Use During Access				Option d) Relevant Phone Use			
	Any Healthcare Provider	Public Providers	Private Providers	Informal Providers	Any Healthcare Provider	Public Providers	Private Providers	Informal Providers	Any Healthcare Provider	Public Providers	Private Providers	Informal Providers	Any Healthcare Provider	Public Providers	Private Providers	Informal Providers
Health-Related Mobile Phone Use[a]	0.115	-0.120	0.212	1.238***	1.135***	1.419***	0.797***	1.444**	0.734**	1.407***	-0.105	0.0327	0.844***	1.524***	0.600**	0.503
	(0.159)	(0.266)	(0.216)	(0.464)	(0.217)	(0.305)	(0.275)	(0.669)	(0.294)	(0.437)	(0.300)	(0.498)	(0.216)	(0.294)	(0.264)	(0.610)
HH Owns ICT and Media Assets	0.253	0.293	0.367	0.520	0.170	0.326	0.278	1.072*	0.229	0.368	0.409	1.209*	0.228	0.399	0.330	1.252*
	(0.270)	(0.271)	(0.330)	(0.844)	(0.250)	(0.254)	(0.312)	(0.582)	(0.262)	(0.241)	(0.318)	(0.643)	(0.265)	(0.244)	(0.312)	(0.662)
Hh. Owns Vehicles	-0.346*	-0.879***	0.259	1.582**	-0.141	-0.666***	0.381**	1.308	-0.229	-0.746***	0.280*	1.141	-0.196	-0.736***	0.328**	1.245
	(0.198)	(0.258)	(0.165)	(0.699)	(0.191)	(0.219)	(0.167)	(0.876)	(0.187)	(0.231)	(0.163)	(0.883)	(0.183)	(0.213)	(0.164)	(0.924)
Country Dummy (1 = Gansu)	-1.100***	-1.787***	-2.177***	-4.248***	-1.294***	-2.251***	-2.287***	-4.849***	-1.171***	-2.349***	-2.151***	-4.555***	-1.239***	-2.359***	-2.284***	-4.770***
	(0.312)	(0.416)	(0.398)	(1.622)	(0.317)	(0.354)	(0.415)	(1.351)	(0.326)	(0.354)	(0.395)	(1.315)	(0.328)	(0.344)	(0.428)	(1.520)
Illness Episode is "Severe"	0.561	0.188	0.435	2.240***	0.333	-0.161	0.403	1.693***	0.487	0.00908	0.419	1.909***	0.442	-0.171	0.389	1.887***
	(0.455)	(0.472)	(0.627)	(0.696)	(0.418)	(0.406)	(0.637)	(0.588)	(0.427)	(0.402)	(0.624)	(0.614)	(0.442)	(0.398)	(0.643)	(0.615)
Gender (1 = Female)	0.0647	-0.214	0.0734	-0.417	-0.0380	-0.362*	0.0740	-0.814	0.0523	-0.337	0.0482	-0.597	0.0230	-0.373*	0.0737	-0.652
	(0.181)	(0.223)	(0.231)	(0.481)	(0.175)	(0.220)	(0.223)	(0.645)	(0.175)	(0.222)	(0.231)	(0.582)	(0.171)	(0.218)	(0.226)	(0.595)
Literacy (1 = Literate)	-0.453*	0.0685	-0.570	-0.703	-0.424*	-0.0207	-0.345	-0.602	-0.466*	-0.110	-0.502	-0.941	-0.451*	-0.0290	-0.387	-0.903
	(0.245)	(0.340)	(0.399)	(1.084)	(0.257)	(0.372)	(0.337)	(0.934)	(0.240)	(0.352)	(0.367)	(0.984)	(0.255)	(0.381)	(0.349)	(0.986)
Highest Completed Grade	0.0190	-0.0160	0.0318	0.0780	0.0371	0.0261	0.00457	0.154	0.0259	0.0375	0.0299	0.108	0.0247	0.0277	0.0117	0.125
	(0.0254)	(0.0368)	(0.0447)	(0.154)	(0.0262)	(0.0369)	(0.0385)	(0.142)	(0.0245)	(0.0378)	(0.0430)	(0.143)	(0.0250)	(0.0382)	(0.0406)	(0.146)
Age Group[b]	0.0682	0.0930	0.220*	0.0483	0.123	0.116	0.187	-0.138	0.102	0.141	0.218*	-0.0899	0.107	0.125	0.205*	-0.0868
	(0.0804)	(0.0928)	(0.116)	(0.208)	(0.0848)	(0.0951)	(0.123)	(0.216)	(0.0820)	(0.0917)	(0.118)	(0.219)	(0.0831)	(0.0946)	(0.123)	(0.215)
Household Asset Index (Quintile)	-0.00910	0.130	0.0225	-0.352**	-0.0597	0.0665	0.00863	-0.498**	-0.0376	0.0928	0.0233	-0.340*	-0.0390	0.0821	0.0188	-0.403*
	(0.0646)	(0.104)	(0.0845)	(0.151)	(0.0675)	(0.0963)	(0.0916)	(0.214)	(0.0643)	(0.0965)	(0.0862)	(0.183)	(0.0653)	(0.0946)	(0.0896)	(0.211)
Household Size	-0.00347	0.0284	-0.00401	0.122	-0.0370	-0.0308	-0.0245	0.124	-0.0182	-0.0196	0.00315	0.0802	-0.0168	-0.0332	-0.0171	0.106
	(0.0310)	(0.0466)	(0.0492)	(0.1000)	(0.0249)	(0.0364)	(0.0524)	(0.119)	(0.0262)	(0.0380)	(0.0511)	(0.125)	(0.0259)	(0.0354)	(0.0518)	(0.128)
Gender (HH Head) (1 = Female)	0.362	0.748**	0.406	2.478**	0.320	0.890***	0.330	2.699**	0.233	0.672**	0.398	2.437*	0.278	0.839**	0.337	2.590*
	(0.237)	(0.353)	(0.389)	(1.083)	(0.236)	(0.339)	(0.401)	(1.336)	(0.225)	(0.321)	(0.399)	(1.325)	(0.229)	(0.332)	(0.403)	(1.344)
Highest Completed Grade (HH Head)	0.0809***	0.0553**	0.0414*	0.184***	0.0667***	0.0362*	0.0432**	0.165**	0.0829***	0.0336	0.0423*	0.214***	0.0804***	0.0321	0.0430*	0.213***
	(0.0195)	(0.0262)	(0.0219)	(0.0653)	(0.0150)	(0.0211)	(0.0218)	(0.0737)	(0.0178)	(0.0222)	(0.0233)	(0.0749)	(0.0177)	(0.0210)	(0.0225)	(0.0768)
Family Members Living Outside Village	-0.176	0.661***	-0.286	0.747	-0.106	0.701***	-0.225	1.122*	-0.130	0.695***	-0.288	0.834	-0.0647	0.792***	-0.186	0.951
	(0.207)	(0.233)	(0.348)	(0.661)	(0.167)	(0.203)	(0.346)	(0.631)	(0.205)	(0.210)	(0.355)	(0.648)	(0.196)	(0.211)	(0.368)	(0.690)
Self-Rated Health[c]	0.210**	0.381***	0.0906	0.702***	0.186**	0.328***	0.145	0.747***	0.220***	0.414***	0.110	0.699***	0.191**	0.316***	0.115	0.752***
	(0.0818)	(0.0957)	(0.131)	(0.243)	(0.0869)	(0.0959)	(0.133)	(0.268)	(0.0829)	(0.0931)	(0.141)	(0.270)	(0.0842)	(0.0955)	(0.135)	(0.288)
Activities of Daily Living (score)[d]	0.321	-0.106	0.747***	1.305***	0.146	-0.227	0.697**	1.142**	0.265	-0.277*	0.693**	1.249**	0.267	-0.247	0.688**	1.202**
	(0.246)	(0.202)	(0.277)	(0.398)	(0.193)	(0.158)	(0.285)	(0.502)	(0.240)	(0.163)	(0.300)	(0.498)	(0.236)	(0.155)	(0.291)	(0.481)
Knows Ambulance Hotline (1 = Aware)	0.135	0.157	0.105	-0.413	0.238	0.395*	0.0645	0.121	0.177	0.284	0.0705	-0.505	0.226	0.415*	0.117	-0.337
	(0.209)	(0.227)	(0.241)	(0.630)	(0.206)	(0.219)	(0.226)	(0.608)	(0.201)	(0.189)	(0.245)	(0.705)	(0.212)	(0.214)	(0.237)	(0.664)
Knows Health Hotline (1 = Aware)	0.107	0.278	0.0642	-0.491	0.145	0.475	0.0575	0.111	0.0675	0.459	0.0798	1.205	0.145	0.475	0.0703	1.008
	(0.241)	(0.522)	(0.316)	(1.541)	(0.260)	(0.470)	(0.318)	(1.327)	(0.258)	(0.494)	(0.312)	(1.129)	(0.250)	(0.490)	(0.309)	(1.144)
Perceived Ambulance Response Time[e]	0.0423	-0.000584	0.117**	0.00474	0.0456	-0.0190	0.104**	-0.135	0.0431	0.0175	0.120**	-0.108	0.0286	-0.00761	0.107**	-0.139
	(0.0457)	(0.0586)	(0.0556)	(0.204)	(0.0457)	(0.0604)	(0.0511)	(0.191)	(0.0462)	(0.0636)	(0.0564)	(0.201)	(0.0457)	(0.0615)	(0.0526)	(0.209)
Distance to Closest Health Provider[f]	0.219*	0.355***	0.256*	0.888**	0.202*	0.314**	0.348**	0.836*	0.212*	0.212*	0.246	0.907**	0.244**	0.289**	0.308**	0.921**
	(0.117)	(0.129)	(0.142)	(0.379)	(0.112)	(0.126)	(0.149)	(0.440)	(0.117)	(0.123)	(0.151)	(0.410)	(0.121)	(0.125)	(0.150)	(0.423)
Resp. Considers Village Clinic / Nurse	-0.0326	-0.536**	0.358**	2.159***	0.00529	-0.376*	0.411**	2.634***	-0.0296	-0.433*	0.343**	2.649***	-0.0203	-0.390*	0.406**	2.677***
	(0.172)	(0.240)	(0.164)	(0.726)	(0.157)	(0.218)	(0.165)	(0.615)	(0.161)	(0.222)	(0.168)	(0.582)	(0.163)	(0.223)	(0.169)	(0.611)
Resp. Considers Small Hospital	0.0874	0.0808	0.351	1.230***	0.0496	0.104	0.342	0.434	0.108	0.136	0.330	1.061**	0.0923	0.0801	0.332	0.938*
	(0.182)	(0.237)	(0.226)	(0.389)	(0.161)	(0.205)	(0.229)	(0.621)	(0.174)	(0.211)	(0.238)	(0.517)	(0.170)	(0.203)	(0.233)	(0.520)
Resp. Considers County Hospital	-0.237	-0.485**	0.0458	0.930	-0.0775	-0.254	0.144	-0.0970	-0.0899	-0.369**	0.00366	0.244	-0.0537	-0.197	0.138	0.222
	(0.181)	(0.208)	(0.159)	(0.737)	(0.159)	(0.159)	(0.180)	(0.829)	(0.175)	(0.181)	(0.187)	(0.698)	(0.163)	(0.165)	(0.191)	(0.715)
Resp. Considers Private Doctor	-0.0884	0.353*	0.0984	-0.853***	-0.0399	0.299*	0.345	-0.444	-0.0165	0.316	0.0929	-0.598*	-0.00566	0.309*	0.286	-0.542
	(0.181)	(0.207)	(0.232)	(0.301)	(0.162)	(0.177)	(0.228)	(0.336)	(0.173)	(0.202)	(0.248)	(0.355)	(0.167)	(0.182)	(0.236)	(0.354)
Resp. Considers Pharmacy	-0.472**	-0.498*	-0.0645	-0.481	-0.627***	-0.735***	-0.149	-0.328	-0.561***	-0.660**	-0.0263	-0.186	-0.607***	-0.783***	-0.155	-0.236
	(0.194)	(0.287)	(0.389)	(0.972)	(0.201)	(0.284)	(0.372)	(0.834)	(0.188)	(0.274)	(0.369)	(0.915)	(0.199)	(0.286)	(0.381)	(0.899)
Resp. Considers Drug Shop	-0.101	-0.177	0.144	-1.921***	-0.113	-0.0288	-0.0432	-2.370***	-0.134	-0.0606	0.0835	-2.240***	-0.140	-0.0299	0.0194	-2.235***
	(0.201)	(0.193)	(0.170)	(0.669)	(0.180)	(0.153)	(0.163)	(0.669)	(0.186)	(0.159)	(0.162)	(0.747)	(0.190)	(0.153)	(0.150)	(0.750)
Resp. Considers Traditional Healer	0.0549	-0.297	0.323	0.591	0.0493	-0.244	0.332	-0.125	0.0127	-0.454**	0.333	0.101	0.0263	-0.323	0.320	0.0440
	(0.178)	(0.208)	(0.236)	(0.826)	(0.168)	(0.209)	(0.233)	(0.887)	(0.169)	(0.187)	(0.230)	(0.880)	(0.169)	(0.212)	(0.233)	(0.891)
Resp. Considers Alternative Medicine	0.0742	0.806	-0.0787	-1.513**	0.0761	1.065	-0.239	-2.290***	0.140	1.022	-0.105	-2.105***	0.171	1.208	-0.173	-2.096***
	(0.479)	(0.755)	(0.692)	(0.636)	(0.491)	(0.835)	(0.745)	(0.773)	(0.465)	(0.774)	(0.685)	(0.701)	(0.464)	(0.795)	(0.751)	(0.700)
Resp. Considers Internet Sources	2.278***	2.344***	0.0580	3.148***	2.527***	2.481***	0.267	2.933***	2.384***	2.235**	0.0633	2.778***	2.490***	2.398***	0.182	2.908***
	(0.884)	(0.894)	(1.316)	(0.860)	(0.888)	(0.898)	(1.477)	(0.844)	(0.893)	(0.900)	(1.287)	(0.979)	(0.876)	(0.902)	(1.438)	(0.915)
Resp. Considers Other Providers	-0.246	0.281	-0.275	-1.117	-0.502*	-0.212	-0.541	-1.711	-0.203	0.239	-0.357	-1.928	-0.226	0.272	-0.357	-1.878
	(0.337)	(0.609)	(0.359)	(1.099)	(0.294)	(0.478)	(0.349)	(1.223)	(0.332)	(0.568)	(0.377)	(1.357)	(0.331)	(0.557)	(0.354)	(1.285)
Constant	-0.289	-0.104	-1.846*	-4.519	-0.344	-0.474	-2.007*	-3.421	-0.403	-0.299	-1.688	-5.571**	-0.637	-0.499	-2.022*	-5.470**
	(0.783)	(1.070)	(1.084)	(2.947)	(0.719)	(1.035)	(1.150)	(2.837)	(0.752)	(1.044)	(1.132)	(2.569)	(0.754)	(1.057)	(1.154)	(2.594)
ln(α)	0.477***	0.597***	0.192**	0.184	0.398***	0.501***	0.171**	0.231	0.458***	0.528***	0.196**	0.273	0.449***	0.502***	0.183**	0.275
	(0.144)	(0.146)	(0.0831)	(0.182)	(0.140)	(0.145)	(0.0815)	(0.192)	(0.145)	(0.144)	(0.0834)	(0.186)	(0.145)	(0.139)	(0.0810)	(0.188)
Number of Observations	592	380	264	81	592	380	264	81	592	380	264	81	592	380	264	81
Pseudo R^2	0.066	0.088	0.098	0.182	0.079	0.102	0.101	0.178	0.069	0.098	0.097	0.171	0.071	0.103	0.099	0.172

Source: Authors.

Notes . Coefficients reported. Village-cluster robust standard errors in parentheses. HH is household.

[a.] Variable specified as per Options a to d. Options a and b involve the same phone use variable for all four types of healthcare access; variables in Options c and d are specific to each type of healthcare access (e.g. phone use during public provider access).

[b.] 1 = "18-24 years," 2 = "25-34 years," 3 = "35-44 years," 4 = "45-59 years," 5 = "60+ years."

[c.] 1 = "very good," 2 = "good," 3 = "moderate," 4 = "bad," 5 = "very bad."

[d.] Computed as average score of seven activities, each coded: 1 = "no difficulty / no assistance," 2 = "mild difficulty / no assistance," 3 = "moderate difficulty / a bit of assistance," 4 = "severe difficulty / a lot of assistance," 5 = "extreme difficulty / cannot do."

[e.] 1 = "< 10 min," 2 = "10-29 min," 3 = "30-59 min," 4 = "60-119 min," 5 = "> 2 hours," 6 = "would not come."

[f.] 1 = "< 10 min," 2 = "10-29 min," 3 = "30-59 min," 4 = "60-119 min," 5 = "> 2 hours."

* p<0.1, ** p<0.05, *** p<0.01

Appendix A3. Full Results of Logit Regression Models to Evaluate "Ideal" Processes of Public Healthcare Access

	Sequence Insensitive	Sequence Sensitive	
	Evaluation a) Any Public Healthcare Access	Evaluation b) Public Healthcare Access With Adherence to Referral	Evaluation c) Public Healthcare Access Without Prior Informal / Private Care
**Household Asset Index (Quintile)**	-0.135* (0.0821)	-0.258** (0.110)	-0.228** (0.102)
**HH Owns ICT and Media Assets**	-0.0653 (0.327)	0.0802 (0.656)	-0.213 (0.537)
**Hh. Owns Vehicles**	0.144 (0.217)	0.200 (0.315)	0.308 (0.288)
**Country Dummy (1 = Gansu)**	0.256 (0.454)	1.737** (0.763)	1.212* (0.650)
**Illness Episode is "Severe"**	1.817*** (0.385)	3.099*** (0.434)	2.704*** (0.402)
**Gender (1 = Female)**	-0.157 (0.202)	0.130 (0.281)	0.0136 (0.258)
**Literacy (1 = Literate)**	-0.149 (0.321)	-0.0527 (0.418)	-0.0271 (0.398)
**Highest Completed Grade**	0.0843** (0.0402)	0.0329 (0.0509)	0.0388 (0.0482)
**Age Group[a]**	-0.00116 (0.0903)	-0.0317 (0.127)	0.0778 (0.117)
**Household Size**	0.0185 (0.0443)	-0.114 (0.0709)	-0.0828 (0.0629)
**Gender (HH Head) (1 = Female)**	-0.351 (0.317)	-0.595 (0.423)	-0.886** (0.407)
**Highest Completed Grade (HH Head)**	-0.0219 (0.0265)	0.0212 (0.0373)	0.0135 (0.0344)
**Family Members Living Outside Village**	-0.125 (0.270)	-0.106 (0.382)	-0.345 (0.357)
**Self-Rated Health[b]**	0.0426 (0.105)	-0.111 (0.129)	-0.0646 (0.122)
**Activities of Daily Living (score)[c]**	-0.218 (0.206)	0.0123 (0.269)	0.284 (0.243)
**Knows Ambulance Hotline (1 = Aware)**	0.147 (0.226)	-0.0360 (0.287)	-0.120 (0.273)
**Knows Health Hotline (1 = Aware)**	0.400 (0.391)	0.256 (0.836)	0.271 (0.694)
**Perceived Ambulance Response Time[d]**	0.0127 (0.0593)	0.00184 (0.0773)	0.0570 (0.0728)
**Distance to Closest Health Provider[e]**	0.0427 (0.119)	0.0181 (0.152)	0.00447 (0.144)
**Resp. Considers Village Clinic / Nurse**	-0.525*** (0.202)	-0.600** (0.274)	-0.712*** (0.252)
**Resp. Considers Small Hospital**	-0.553*** (0.184)	-0.547** (0.240)	-0.465** (0.228)
**Resp. Considers County Hospital**	-0.0738 (0.203)	-0.266 (0.270)	-0.0173 (0.253)
**Resp. Considers Private Doctor**	0.548*** (0.210)	-0.600** (0.258)	-0.669*** (0.242)
**Resp. Considers Pharmacy**	0.482* (0.252)	0.944*** (0.278)	1.017*** (0.274)
**Resp. Considers Drug Shop**	-0.296 (0.242)	-0.934*** (0.317)	-1.048*** (0.308)
**Resp. Considers Traditional Healer**	-0.518** (0.257)	-0.0156 (0.549)	-0.259 (0.431)
**Resp. Considers Alternative Medicine**	0.717 (1.384)	-1.538 (1.685)	-1.547 (1.560)
**Resp. Considers Internet Sources**	-0.163 (0.828)	0.230 (0.825)	0.256 (0.809)
**Resp. Considers Other Providers**	-0.794 (0.530)		-2.277* (1.346)
**Constant**	-0.468 (0.994)	-0.926 (1.890)	-1.166 (1.601)
**Number of Observations**	669	649[f]	669
**Pseudo R^2**	0.093	0.320	0.270

Source: Authors.

Notes . Coefficients reported. Standard errors in parentheses. HH is household.

a. 1 = "18-24 years," 2 = "25-34 years," 3 = "35-44 years," 4 = "45-59 years," 5 = "60+ years."

b. 1 = "very good," 2 = "good," 3 = "moderate," 4 = "bad," 5 = "very bad."

c. Computed as average score of seven activities, each coded: 1 = "no difficulty / no assistance," 2 = "mild difficulty / no assistance," 3 = "moderate difficulty / a bit of assistance," 4 = "severe difficulty / a lot of assistance," 5 = "extreme difficulty / cannot do."

d. 1 = "< 10 min," 2 = "10-29 min," 3 = "30-59 min," 4 = "60-119 min," 5 = "> 2 hours," 6 = "would not come."

e. 1 = "< 10 min," 2 = "10-29 min," 3 = "30-59 min," 4 = "60-119 min," 5 = "> 2 hours."

f. Variable predicts failure perfectly. Variable dropped and 20 observations not used.

* p<0.1, ** p<0.05, *** p<0.01
